# Rheumatoid arthritis concurrent with striatal hand deformity

**DOI:** 10.1093/rap/rkac068

**Published:** 2022-08-19

**Authors:** Takashi Nawata, Masafumi Yano

**Affiliations:** Department of Medicine and Clinical Science, Yamaguchi University Graduate School of Medicine, Ube, Japan; Department of Medicine and Clinical Science, Yamaguchi University Graduate School of Medicine, Ube, Japan

A 63-year-old woman presented with polyarthralgia and stiffness of the right wrist joint. She had been diagnosed with focal dystonia 8 years prior. Her right wrist joint showed rigidity, and was diagnosed as striatal hand owing to dystonia. Four months earlier, she became aware of polyarthralgia and reduced mobility of the right wrist joint. Her attending physician diagnosed it as a worsening of the striatal hand deformity, and given that she also had swollen joints, she consequently visited our hospital. Laboratory examinations showed positivity of RF (139.6 IU/ml) and anti-CCP antibodies (13.2 U/ml). Her right wrist joint showed signs of bony ankylosis (Fig. 1A, B); however, CT and hand radiography showed no signs of this condition (Fig. 1C, D). Findings suggestive of psoriasis were not found. She was diagnosed with RA, and oral MTX and prednisolone were administered. Polyarthralgia, rigidity and mobility of the right wrist improved after treatment (Fig. 1E, F).

**Figure 1.**
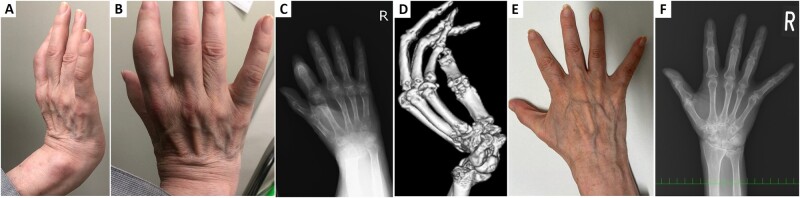
Clinical and imaging findings from the patient. **(A, B)** The patient’s finger joints were swollen, and the right wrist joint showed signs of bony ankylosis. **(C, D)** Hand radiography showed scattered osteopenia in the bones, dorsal dislocation of the distal ulnar joint, radiocarpal joint space narrowing, and flexion of index finger DIP joint. However, CT and hand radiography did not reveal bony ankylosis. **(E, F)** The patient’s finger joints and hand radiographic findings improved after treatment

Striatal hand deformity is observed in parkinsonian disorders [1]. Many cases of RA mimicking striatal hand deformity have been documented [2]. In contrast, RA concurrent with striatal hand is a rare presentation. Our case emphasizes the importance of recognizing that striatal hand deformity can be accompanied by RA.

## Data Availability

The authors declare that all relevant data supporting the findings of this case report are available within the article.
